# Characteristic of TIGIT and DNAM-1 Expression on Foxp3+ *γδ* T Cells in AML Patients

**DOI:** 10.1155/2020/4612952

**Published:** 2020-07-27

**Authors:** Zhenyi Jin, Wanyi Ye, Tianbi Lan, Yun Zhao, Xiaxin Liu, Jie Chen, Jing Lai, Shaohua Chen, Xueyun Zhong, Xiuli Wu

**Affiliations:** ^1^Institute of Hematology, School of Medicine, Key Laboratory for Regenerative Medicine of Ministry of Education, Jinan University, Guangzhou 510632, China; ^2^Integrated Chinese and Western Medicine Postdoctoral Research Station, Jinan University, Guangzhou 510632, China; ^3^Department of Pathology, School of Medicine, Jinan University, Guangzhou 510632, China; ^4^Department of Hematology, First Affiliated Hospital, Jinan University, Guangzhou 510632, China

## Abstract

Foxp3+ *γδ* regulatory T (*γδ* Treg) cells promote tumor growth by various mechanisms and induce immuno-senescence. The novel immune checkpoint coinhibitory receptor T cell Ig and ITIM domain (TIGIT) shares similar ligands as the costimulatory receptor DNAX accessory molecule 1 (DNAM-1) and suppresses T cell responses in tumor patients. This study is aimed at characterizing whether the TIGIT/DNAM-1 axis is involved in the distribution and expression of Foxp3+ *γδ* Treg cell subsets in acute myeloid leukemia (AML) patients of different clinical statuses: *de novo* AML (27 patients), AML in nonremission (NR) (7 patients), and AML in complete remission (CR) (12 patients). Our data demonstrated that the proportions of Foxp3+, TIGIT+Foxp3+, and DNAM-1+Foxp3+ *γδ* T cells are significantly higher in *de novo* and NR patients. High levels of TIGIT and DNAM-1 on Foxp3+ *γδ* T cells correlated with increased Foxp3+ *γδ* T cell frequencies. In addition, a high TIGIT/DNAM-1 ratio was observed in *de novo* AML patients and healthy individuals (HIs). Furthermore, the phenotypic abnormalities in Foxp3+, TIGIT+Foxp3+, and DNAM-1+Foxp3+ *γδ* T cells were restored when the patients achieved CR after chemotherapy. Moreover, higher TIGIT+Foxp3+ *γδ* T cells were associated with AML patients who had poor overall survival and were an independent risk factor for prognosis. In conclusion, our study reveals for the first time that the TIGIT/DNAM-1 axis may be involved in Foxp3+ *γδ* Treg cells and indicates the clinical progression and prognosis of AML patients of different clinical statuses, which is considered beneficial for efficient AML immunotherapy.

## 1. Introduction

Acute myeloid leukemia (AML) is the most common type of myeloid malignancy in adults, and with the exception of the acute promyelocytic leukemia (APL, M3) subtype, this disease has an overall poor prognosis with limited change in the standard of care [1, 2]. In leukemic environments, regulatory T cells (Tregs) contribute to immune escape by suppressing antileukemia activity, and these cells include multiple T cell subsets that control the immune response through a variety of mechanisms [3]. Tregs, which express the transcription factor Foxp3, are characterized by their high immunosuppressive function for maintaining self-tolerance and regulating the innate and adaptive immune systems [4]. Elevated expression of Tregs in the tumor microenvironment has been reported for hematological malignancies, and this has been correlated with progression and poor survival. Cosignalling receptors, including costimulatory and coinhibitory receptors, play an indispensable role in regulating the immune response [5]. Coinhibitory receptors, such as programmed cell death protein-1 (PD-1) and cytotoxic T lymphocyte associated-4 (CTLA-4), have been reported to correlate with Treg dysfunction and participate in evading immune surveillance [6–8]. Conversely, costimulatory receptors such as CD28, OX40, and 4-1BB have also been widely implicated in T cell stimulation and immune homeostasis [9]. Among these inhibitory receptors, T cell Ig and ITIM domain (TIGIT), a novel immune inhibitory receptor, has been reported to be expressed not only by Tregs but also by activated T and NK cells. TIGIT, a member of the immunoglobulin receptor superfamily, consists of an immunoglobulin variable (IgV) region-like domain, a type I transmembrane domain, and a cytoplasmic tail, including an immunoreceptor tyrosine-based inhibitory motif (ITIM) and an immunoglobulin tail tyrosine (ITT)-like motif [10]. Several lines of evidence support the role of TIGIT in regulating Treg-mediated suppression and have demonstrated that the TIGIT locus is hypomethylated in human Treg cells, which bind to Foxp3 [11]. Interestingly, TIGIT competes with the costimulatory receptor DNAX accessory molecule 1 (DNAM-1, also known as CD226) for binding to the same ligands poliovirus receptor (CD155, PVR) and nectin-2 (CD112, PVRL2) [12]. TIGIT can act in Tregs to augment their suppression, while DNAM-1 disrupts their suppression and stability [13].

*γδ* T cells represent a small T cell population in human peripheral blood (PB) and share characteristics of the innate and adaptive immune responses. In addition, *γδ* T cells play crucial roles among the immune cell subsets in the tumor microenvironment; however, various conflicting functions have been described [14]. A new regulatory subset of *γδ* T cells that express Foxp3, termed *γδ* regulatory T cells (*γδ* Tregs), has been confirmed to be at a low frequency in tumor-infiltrating leukocytes (TILs) and human PB [15, 16]. Similar to conventional Tregs, inhibitory receptors are expressed on *γδ* Tregs, and the mechanisms by which their suppressive activity is mediated have been reported [14, 17]. A previous study identified specific characteristics of suppressive *γδ* T cells that differentiate them from Tregs as well as their molecular mechanisms responsible for suppressive functions in healthy adult donors [18]. *γδ* Tregs exert their suppressive properties through a host of tolerogenic enzymatic pathways and cytokines and the expression of multiple inhibitory receptors [19]. Recent research has reported that *γδ* T cells exhibit an exhausted state through PD-1 upregulation in AML patients at diagnosis, and whether there is correlation with other immune checkpoint proteins, such as TIGIT and *γδ* Treg cells in AML remains unclear [20]. Here, we determined the characteristics of the paired receptors TIGIT and DNAM-1 on Foxp3+ *γδ* Treg cells in AML patients with different clinical statuses and discuss their influence on clinical outcome.

## 2. Materials and Methods

### 2.1. Samples

PB samples were obtained from 27 newly diagnosed AML patients, including 14 males and 13 females (median age: 62 years, range: 18-88 years). Seven cases had AML in nonremission (NR), including 5 males and 2 females (median age: 66 years, range: 35-90 years), and 12 cases had AML in complete remission (CR), including 5 males and 7 females (median age: 50.5 years, range: 24-78 years). There were eight pairs of pre- and postchemotherapy samples among these patients. The clinical data of the patients are listed in Supplemental Tables 1 and 2. PB from 21 healthy individuals (HIs), including 11 males and 10 females (median age: 57 years, range: 25-83 years), were used as controls.

### 2.2. Reagents

Cell surface staining for flow cytometry was performed using the antibodies CD3-APC-Cy7 (Clone: SK7), *γδ*-PE-Cy7 (Clone: B1), TIGIT-BV421 (Clone: A15153G), and DNAM-1-PE (Clone: 11A*δ*) together with a BV421 isotype Control (Clone: G155-178) and a PE isotype Control (Biolegend, San Diego, USA). The Foxp3-Alexaflour 647 (Clone: 236A/E7) fluorescent antibody was stained independently.

### 2.3. Cell Surface and Intracellular Cytokine Staining

A total of 300 *μ*L PB was collected in a tube, and 5 *μ*L per sample was incubated with each antibody except Foxp3-Alexaflour 647 for 20 min at room temperature in the dark. Erythrocytes were lysed using RBC Lysis Buffer (BD, Biosciences, USA) for 10 min in the dark. After completely washing the cells, they were fixed and permeabilized with the Foxp3 Fix/Perm Buffer Set (Biolegend, San Diego, USA) according to the manufacturer's instructions. Following incubation with the Foxp3 mAb for 30 min, cells were washed and resuspended with 1X Flow Cytometry Perm Buffer for analysis by flow cytometry. All samples were analyzed with a BD Verse flow cytometer (BD, Biosciences, USA), and FlowJo software was used to analyze data.

### 2.4. Statistical Analysis

All data were represented as medians. Statistical differences between two groups were analyzed by the Mann–Whitney *U* test, and statistical differences among three groups were analyzed by the Kruskal-Wallis test. For paired samples, the Wilcoxon signed-rank test was used for comparison. Spearman's rank coefficient was used to analyze correlations. Overall survival (OS) time was defined as the time from a new diagnosis to the survival time or death from any cause. The Kaplan-Meier method and multivariate Cox regression analysis were used to analyze between-group survival differences. All statistical tests were 2-tailed. *P* values less than 0.05 were considered statistically significant (^∗^*P* < 0.05 and ^∗∗^*P* < 0.01). All calculations were performed using GraphPad Prism 7.0 software (GraphPad Software Inc., San Diego, CA) and SPSS 10.0.

## 3. Results

### 3.1. TIGIT Is Enriched on *γδ* Tregs in AML Patients

**Figure 1:**
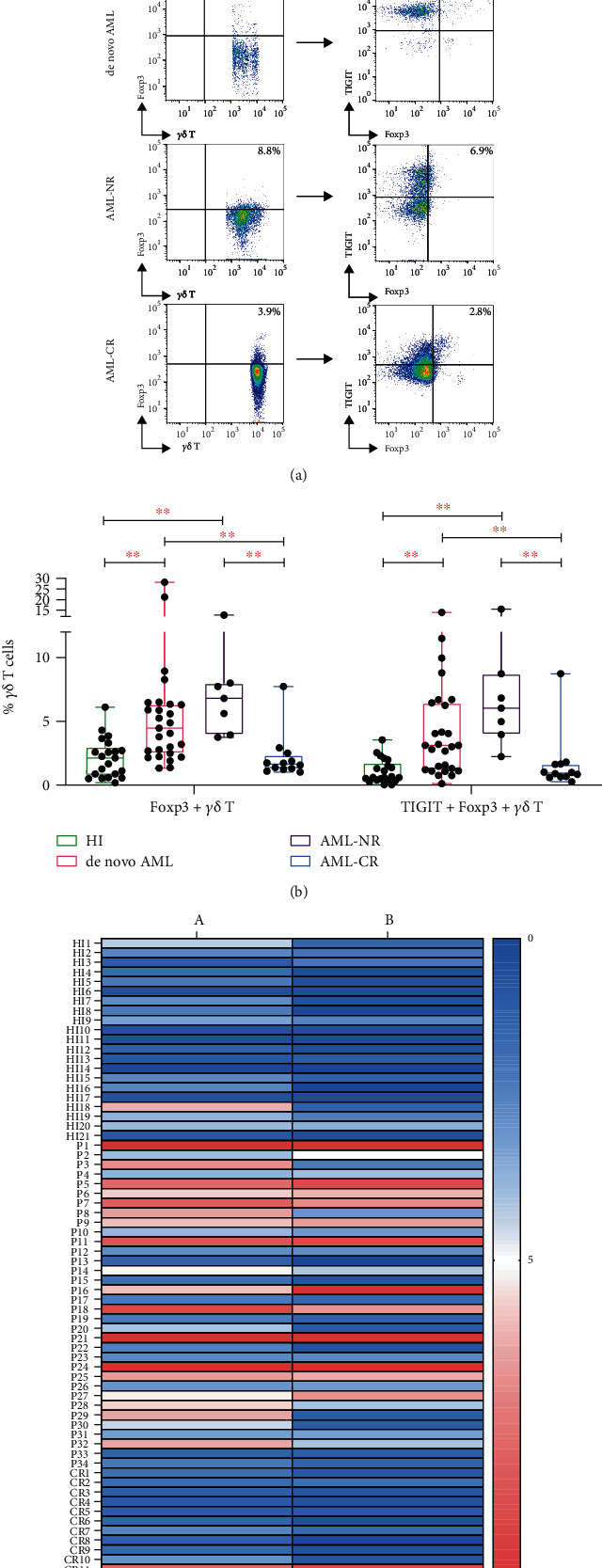
Expression of Foxp3+ and TIGIT+Foxp3+ *γδ* T cells from AML patients and HIs. (a) The expression of Foxp3+ and TIGIT+Foxp3+ *γδ* T cells from a healthy individual (HI), a *de novo* AML patient, an AML patient in nonremission (AML-NR), and an AML patient in complete remission (AML-CR). (b) Comparison of the percentage of Foxp3+ and TIGIT+Foxp3+ *γδ* T cells from AML patients and HIs. (c) Heatmap representing the frequency of Foxp3+ and TIGIT+Foxp3+ *γδ* T cells in AML patients and HIs. a: Foxp3+ *γδ* T cells; b: TIGIT+Foxp3+ *γδ* T cells.

Our initial studies examined the frequency of Foxp3+ *γδ* T cells in AML patients and healthy controls. We found that the proportion of Foxp3+ *γδ* T cells was higher in both *de novo* AML and AML-NR patients compared with AML-CR patients and HIs (NR vs. CR, *P* ≤ 0.001; NR vs. HI, *P* ≤ 0.001; *de novo* vs. CR, *P* ≤ 0.001; *de novo* vs. HI, *P* ≤ 0.001) with the observed pattern AML-NR (median: 6.81%, range: 3.75%-12.80%) > *de novo* AML (median: 4.47%, range: 1.33%-28.20%) > HIs (median: 2.13%, range: 0.18%-6.12%) > AML-CR (median: 1.65%, range: 1.02%-7.74%) groups (Figures 1(a)–1(c)). We next wanted to assess TIGIT expression on Foxp3+ *γδ* T cells in the AML groups and HIs. Similar differences and patterns were found with AML-NR (median: 6.03%, range: 2.24%-15.50%) > *de novo* AML (median: 3.11%, range: 0.11%-14.00%) > AML-CR (median: 0.95%, range: 0.26%-8.73%) > HIs (median: 0.60%, range: 0.03%-3.55%; NR vs. CR, *P* = 0.001; NR vs. HI, *P* ≤ 0.001; *de novo* vs. CR, *P* = 0.004; *de novo* vs. HI, *P* ≤ 0.001) (Figures 1(a)–1(c)).

### 3.2. Increased Expression of DNAM-1 on Foxp3+ *γδ* Tregs in AML Patients

**Figure 2:**
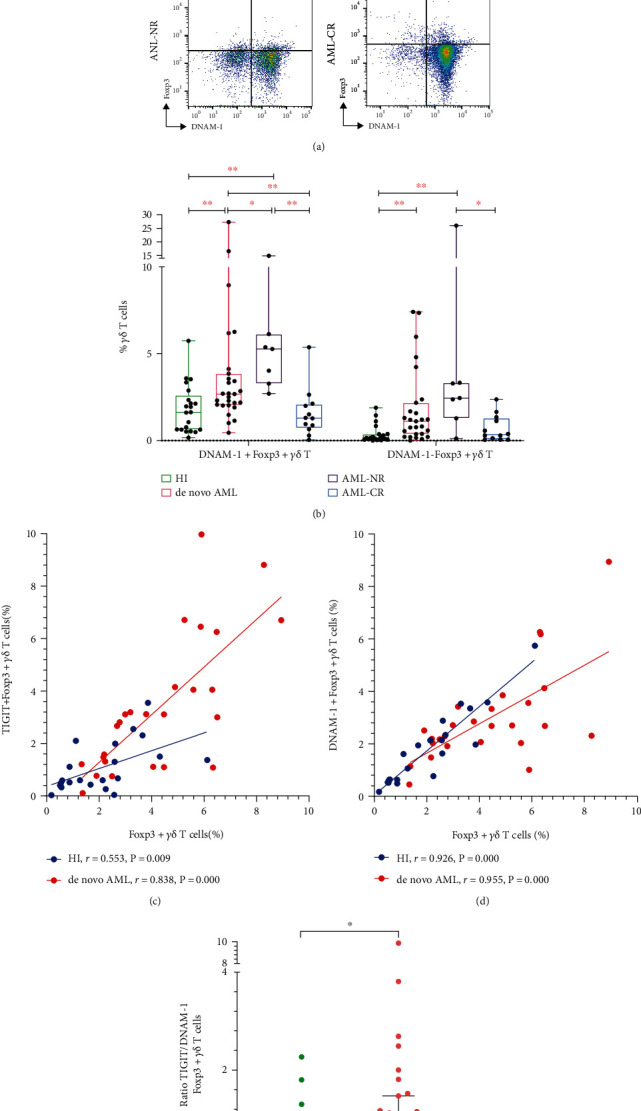
Expression of DNAM-1 on Foxp3+ *γδ* T cells and correlation between TIGIT and DNAM-1 on Foxp3+ *γδ* T cells. (a) The expression of DNAM-1 on Foxp3+ *γδ* T cells from a HI, a *de novo* AML patient, an AML-NR patient, and an AML-CR patient. (b) Comparison of DNAM-1 on Foxp3+ *γδ* T cells from AML patients and HIs. (c) Correlation between the frequency of TIGIT+Foxp3+ and Foxp3+ *γδ* T cells in *de novo* AML patients compared with HIs. (d) Correlation between the frequency of DNAM-1+Foxp3+ and Foxp3+ *γδ* T cells in *de novo* AML patients compared with HIs. (e) Comparison of the ratio of TIGIT to DNAM-1 (TIGIT/DNAM-1) expression in Foxp3+ *γδ* T cells from *de novo* AML patients and HIs.

We next investigated the frequency and expression pattern of DNAM-1 on Foxp3+ *γδ* T cells and found a higher level of DNAM-1 expression in the AML-NR and *de novo* AML groups compared to the AML-CR and HI groups with the pattern AML-NR (median: 5.27%, range: 2.70%-14.90%) > *de novo* AML (median: 2.68%, range: 0.45%-27.30%) > HI (median: 1.63%, range: 0.17%-5.74%) > AML-CR (median: 1.30%, range: 0.04%-5.37%; NR vs. *de novo*, *P* = 0.035; NR vs. CR, *P* ≤ 0.001; NR vs. HI, *P* ≤ 0.001; *de novo* vs. CR, *P* = 0.002; *de novo* vs. HI, *P* = 0.005) (Figures 2(a) and 2(b)). Similarly, an increased frequency of DNAM-1-Foxp3+ *γδ* T cells was observed in the AML-NR and *de novo* AML groups compared to the AML-CR and HI groups with the pattern AML-NR (median: 2.45%, range: 0.12%-26.00%) > *de novo* AML (median: 1.11%, range: 0.001%-7.41%) > AML-CR (median: 0.34%, range: 0.04%-2.37%) > HI (median: 0.15%, range: 0.001%-1.89%; NR vs. CR, *P* = 0.010; NR vs. HI, *P* = 0.001; *de novo* vs. HI, *P* ≤ 0.001) (Figures 2(a) and 2(b)).

### 3.3. Relevance of TIGIT and DNAM-1 Expression on Foxp3+ *γδ* Treg Cells

We further investigated the correlation between the frequency of TIGIT and DNAM-1 on Foxp3+ *γδ* T cells in the *de novo* AML and HI groups. Interestingly, the frequency of TIGIT+Foxp3+ *γδ* T cells was positively correlated with Foxp3+ *γδ* T cells in *de novo* AML patients and HIs (*P* ≤ 0.001, *r* = 0.838, and *P* = 0.009, *r* = 0.553, respectively) (Figure 2(c)). In addition, there was also a positive correlation between the percentages of DNAM-1+Foxp3+ *γδ* T cells and Foxp3+ *γδ* Treg cells in the *de novo* AML and HI groups (*P* ≤ 0.001, *r* = 0.955, and *P* ≤ 0.001, *r* = 0.926, respectively) (Figure 2(d)). Based on the increased expression trend for TIGIT and DNAM-1 on Foxp3+ *γδ* T cells, we next assessed the ratio of TIGIT to DNAM-1 (TIGIT/DNAM-1) expression in Foxp3+ *γδ* T cells in the *de novo* AML and HI groups. Notably, we found a high ratio of TIGIT/DNAM-1 expression in Foxp3+ *γδ* T cells in *de novo* AML patients (median: 1.00) compared to HIs (median: 0.69) (*P* = 0.040) (Figure 2(e)).

### 3.4. Distribution and Influence of TIGIT and DNAM-1 on Foxp3+ *γδ* Treg T Cell Subsets in AML Patients after Treatment and Clinical Outcomes

**Figure 3:**
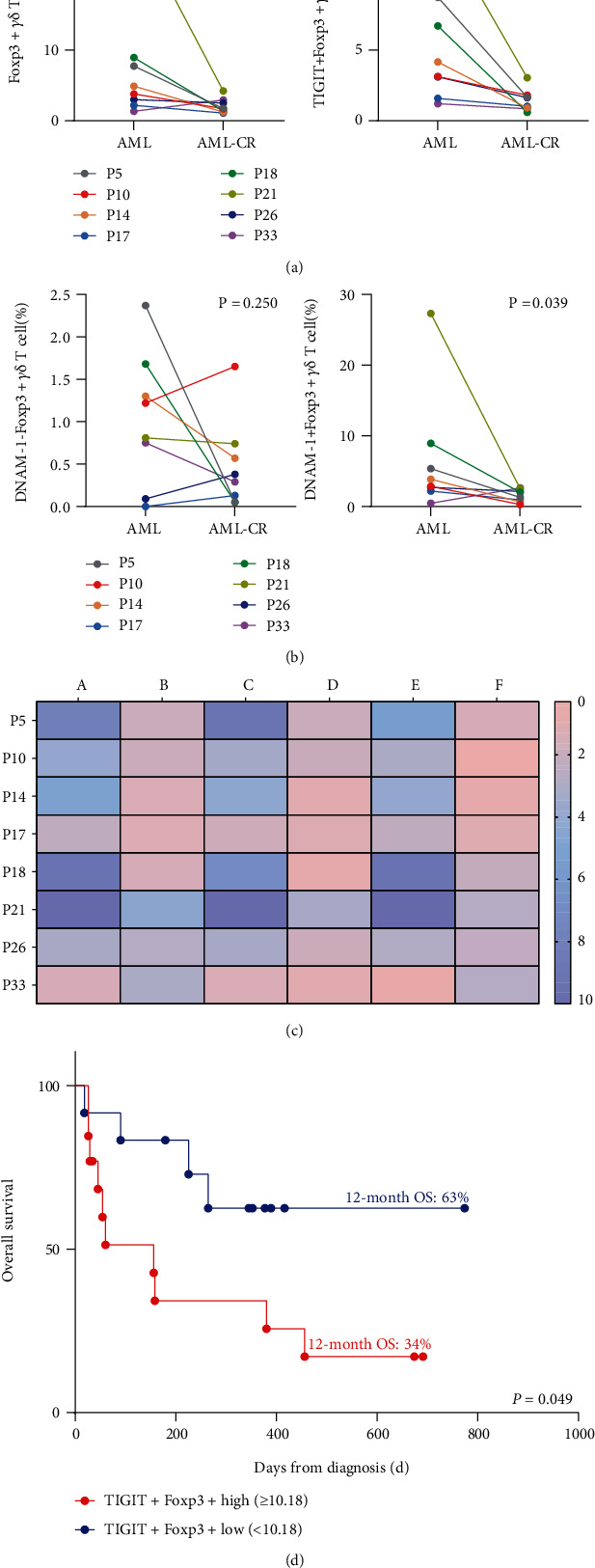
Dynamic change and survival analysis of TIGIT and DNAM-1 on Foxp3+ *γδ* T cells in AML patients. (a) Pairwise comparisons of Foxp3+ and TIGIT+Foxp3+ *γδ* T cells in eight AML patients who achieved CR. (b) Pairwise comparisons of Foxp3+ and DNAM-1 on Foxp3+ *γδ* T cells in eight AML patients who achieved CR. (c) Summary of the dynamic changes in the Foxp3+, TIGIT+Foxp3+, and DNAM-1+Foxp3+ *γδ* T cell subsets in AML patients before and after treatment. a: Foxp3+ *γδ* T cells before treatment; b: Foxp3+ *γδ* T cells after treatment; c: TIGIT+Foxp3+ *γδ* T cells before treatment; d: TIGIT+Foxp3+ *γδ* T cells after treatment; e: DNAM-1+Foxp3+ *γδ* T cells before treatment; f: DNAM-1+Foxp3+ *γδ* T cells after treatment. (d) Comparison of OS curves of *de novo* AML patients based on TIGIT+Foxp3+ *γδ* T cell expression. The red line indicates high TIGIT+Foxp3+ expression; the blue line indicates low TIGIT+Foxp3+ expression.

TIGIT and DNAM-1 play opposing roles in the regulation of immune function; thus, we further sought to determine how they influence *γδ* Treg cell subsets by treatment. Paired comparisons of the percentage of TIGIT and DNAM-1 on Foxp3+ *γδ* T cells were conducted for eight patients before and after induction chemotherapy. The results demonstrated that Foxp3+ *γδ*, TIGIT, and DNAM-1 on Foxp3+ *γδ* cell subsets were evidently decreased in AML patients who achieved CR (Foxp3+ *γδ*: *P* = 0.039; TIGIT+Foxp3+ *γδ*: *P* = 0.008; DNAM-1+Foxp3+ *γδ* T cells: *P* = 0.039), which may be due to the elimination of blasts. There were no significant differences in the frequency of DNAM-1-Foxp3+ *γδ* T cells (*P* = 0.250) (Figures 3(a)–3(c)).

To further analyze correlations between TIGIT+Foxp3+ and DNAM-1+Foxp3+ *γδ* T cells, clinical outcome, and OS, we next divided the subsets into high-expression and low-expression groups based on median proportions for 27 *de novo* AML patients and assessed their associations. Strikingly, we observed that AML patients with high expression of TIGIT+Foxp3+ *γδ* T cell subsets correlated with poor OS (TIGIT+Foxp3+^high^ vs. TIGIT+Foxp3+^low^, 12-month OS 34% vs. 63%, *P* = 0.049) (Figure 3(d)). Moreover, multivariate Cox regression analysis indicated that the frequency of TIGIT+Foxp3+ *γδ* T cells was an independent prognostic risk factors for AML prognosis (hazard ratio: 1.142, 95% confidence interval: 0.976-1.336).

## 4. Discussion

It is well known that T cells with the exhausted and suppressive phenotype may have reduced activation and be related to poor prognosis for AML patients [21, 22]. Elucidation of the role of TIGIT on *γδ* Tregs in this context may assist in understanding the dysfunctional immunobiology of *γδ* cells in AML patients of different clinical statuses. Under normal physiological conditions, *γδ* Treg cells exist at low frequency in PB, and deficiencies in the *γδ* Treg frequency and function have been associated with autoimmune diseases [16]. In our investigation, we examined the TIGIT expression on Foxp3+ *γδ* T cells in AML patients of different clinical statuses for the first time and observed that the proportions of Foxp3+ *γδ* T cells and TIGIT+Foxp3+ *γδ* T cells were increased in both *de novo* AML and AML-NR patients. Hence, our results are in line with the suggestion of an increased frequency of TIGIT being mainly concentrated on Foxp3+ *γδ* T cells, which potentially correlates with the progression of AML patients, resulting in functional impairment of *γδ* T cells. A prior study suggested that TIGIT expression was maintained by Foxp3 and played an essential role in immune regulation [16]. Our data suggest that TIGIT signalling might also be involved in the regulation of Foxp3+ *γδ* T cells in patients with AML.

Previously, it is reported that DNAM-1 is upregulated on the majority of thymic-derived Tregs after activation and might play an essential role in IL-10-producing Tregs in healthy donors [23]. To further investigate the TIGIT/DNAM-1 axis in AML patients, we further examined DNAM-1 expression on *γδ* Treg subsets and found that the expression of DNAM-1 was largely concentrated in *γδ* Treg populations in AML-NR and *de novo* patients, which may be related to T cell activation status. A prior study reported that there was divergent DNAM-1 expression in Tregs and CD4+ effector T cells in melanoma patient TILs, demonstrating that different mechanisms are involved in regulating DNAM-1 expression in different functional T cell subsets [13]. The mechanism behind this phenomenon is not entirely understood and needs further assessment. Interestingly, we found a positive correlation between the increased TIGIT or DNAM-1 expression of Foxp3+ *γδ* Treg cells and Foxp3+ *γδ* Treg cells in AML patients. In addition, a high TIGIT/DNAM-1 ratio in Foxp3+ *γδ* T cells in AML patients was also found, which indicates that the increased trend in TIGIT expression was predominant in the PB of AML patients. Consistent with a previous study, a high TIGIT/DNAM-1 ratio in Tregs could also be found in melanoma patients, and this correlated with poor clinical outcome, which could promote Treg stability and its suppressive function.

Despite the increased insight into the phenotype of Foxp3+ *γδ* Treg cells, whether these cells correlate with clinical outcome remains poorly understood [17, 24]. Our previous findings have shown that clonally expanded V*δ*4+ and V*δ*8+ T cells might contribute to the immune response in leukemia, while clonally expanded V*δ*5+ and V*δ*6+ T cells might be related to AML disease progression [25, 26]. Thus, further characterization of the heterogeneity of *γδ* Treg cells in leukemia may help to better understand the T cell dysfunction and immune status in patients with AML. Our observation of dynamic changes contrasts with the alterations in TIGIT and DNAM-1 expression on *γδ* Tregs in AML patients. Foxp3+, TIGIT+Foxp3+, and DNAM-1+Foxp3+ *γδ* T cells were decreased in AML patients who achieved CR, indicating a potentially distinct role for the TIGIT/DNAM-1 axis in the regulation of *γδ* Tregs. These new findings have emerged from our data that dynamic changes in *γδ* Treg subsets occur following chemotherapy with therapeutic responses, which may reflect immune reconstitution. However, further investigation of a large cohort of samples is needed to evaluate the predictive value. In addition, we found that we can evaluate AML prognosis based on the frequency of TIGIT/DNAM-1 expression on Foxp3+ *γδ* Treg cells. Overall, a high level of TIGIT+Foxp3+ *γδ* T cells is associated with poor OS of AML patients; thus, we will further confirm the function of these subsets and whether they could be a potential immune biomarker for adverse prognosis.

## 5. Conclusions

In summary, we characterized for the first time the frequency and expression pattern of TIGIT and DNAM-1 on Foxp3+ *γδ* Treg cells in AML patients of different clinical statuses. Our findings demonstrated that Foxp3+ *γδ* T cells express a high-level of TIGIT and DNAM-1 and a high TIGIT/DNAM-1 ratio in Foxp3+ *γδ* T cells, which leads to better understanding of the TIGIT/DNAM-1 axis on *γδ* Treg cells in AML patients. In addition, significantly increased TIGIT+Foxp3+ or DNAM-1+Foxp3+ *γδ* T cell subsets were restored in AML patients who achieved CR after chemotherapy. Moreover, TIGIT+Foxp3+ *γδ* T cells were associated with poor clinical outcomes, which might be potential biomarker. However, more *in vivo* experiments are required to elucidate the function of the TIGIT+Foxp3+ and DNAM-1+Foxp3+ *γδ* T cell subsets, and whether these cells could serve as a target for immunotherapy should be investigated.

## Data Availability

The datasets used and/or analyzed during the current study are available from the corresponding author upon reasonable request.

## Abbreviations

AML: Acute myeloid leukemia
APL: Acute promyelocytic leukemia
Tregs: Regulatory T cells
PD-1: Programmed cell death protein-1
CTLA-4: Cytotoxic T lymphocyte associated-4
TIGIT: T cell Ig and ITIM domain
DNAM-1: DNAX accessory molecule 1
PB: Peripheral blood
*γδ* Tregs: *γδ* Regulatory T cells
TILs: Tumor-infiltrating leukocytes
NR: Nonremission
CR: Complete remission
HIs: Healthy individuals
OS: Overall survival.

## Supplementary Materials

Supplementary Materials Supplementary Table 1: the characteristic of AML patients and healthy individuals. Supplementary Table 2: the clinical information of de novo AML patients.
