# Supplementary material for: Characteristic of TIGIT and DNAM-1 Expression on Foxp3+ γδ T Cells in AML Patients
Source: Biomed Res Int. 2020 Jul 27;2020:4612952. doi: 10.1155/2020/4612952 (PMC7403925; doi:10.1155/2020/4612952)
Supplement: Supplementary Materials — Supplementary Table 1: the characteristic of AML patients and healthy individuals. Supplementary Table 2: the clinical information of de novo AML patients. [file 4612952.f1.pdf]

Supplementary Table 1: The characteristic of AML patients and healthy individuals

Factor	AML-NR	*de novo* AML	AML-CR	HI
The number of the case	7	27	12	21
Age (median; range)	66 (35-90)	62 (18-88)	50.5 (24-78)	57 (25-83)
Gender (Male / Female)	(5/2)	(14/13)	(5/7)	(11/10)
Clinical index				
WBC (10^9/L) (median; range)	4.3 (0.3-29.4)	6.0 (1.0-132.7)	4.8 (2.1-9.9)	6.2 (4.4-8.5)
PLT (10^9/L) (median; range)	24.0 (1.0-248.0)	40.0 (1.0-632.0)	152.3 (12.0-555.0)	226.0 (97.0-300.0)
RBC (10^9/L) (median; range)	2.3 (1.9-2.8)	2.2 (1.1-3.9)	3.3 (1.9-4.4)	4.9 (4.1-5.9)
Hb (g/L) (median; range)	59.0 (8.0-71.0)	70.0 (48.0-116.0)	93.5 (61.0-138.0)	145.0 (91.0-165.0)
Blast (%) (median; range)	80 (29-93)	44 (21-95)	0 (0-5)	/
FAB classification				
M1	0	1	1	/
M2	0	5	2	/
M3	1	5	5	/
M4	0	2	1	/
M5	4	8	1	/
Undetermined	2	6	2	/
Therapy				/
Chemotherapy	7	27	12	/
Transplantation	0	1	0	/

Notes: WBC: white blood cell, RBC: red blood cell, PLT: platelet, Hb: hemoglobin, F: female, M: male, CR: complete remission, NR: no remission

Supplementary Table 2: The clinical information of *de novo* AML patients

Case No.	Disease status	Subtype	Genotype	Chemotherapy
P8	*de novo*	M5	ND	SC
P9	*de novo*	M2	ND	Azacitidine
P10	*de novo*	M1	FLT3-ITD, CEBPA	DHA
P11	*de novo*	M5	DNMT3A, IDH2, SRSF2, RUNX1	MA
P12	*de novo*	M3	ND	Refused therapy
P13	*de novo*	M2b	AML1-ETO	SC
P14	*de novo*	M3	FLT3-ITD, PML-RARα	ATRA+ATO
P15	*de novo*	M2	K-RAS, DEK-CAN, EA2-HLF	DCAG
P16	*de novo*	Undetermined	AML1-ETO, TET2, RUNX1-RUNX1T1	IA
P17	*de novo*	M2	MLL-ELL	DA
P18	*de novo*	M3	PML-RARα	ATRA+ATO
P19	*de novo*	Undetermined	TP53	DCAG
P20	*de novo*	M5	MLL-AF9, EVI1	IA
P21	*de novo*	M4	ND	IDA+ Azacitidine
P22	*de novo*	M3	PML-RARα	ATRA+ATO
P23	*de novo*	M5	FLT3-ITD, CEBPA	IA
P24	*de novo*	M5	ATM, JAK2, CXCR4	Ara-C+DCA
P25	*de novo*	M5	FLT3	IA
P26	*de novo*	Undetermined	ND	IA
P27	*de novo*	Undetermined	NPM1	DA
P28	*de novo*	Undetermined	ND	Refused therapy
P29	*de novo*	Undetermined	NRAS, TP53, STAG2	IA
P30	*de novo*	M5	ND	SC
P31	*de novo*	M4	TET2, SF3B1	DCAG
P32	*de novo*	M2	ND	DA
P33	*de novo*	M3	PML-RARα	ATRA+ATO
P34	*de novo*	M5	ND	IA

Notes: ND: not detected; DHA: daunorubicin+ homoharringtonine + cytarabine; MA: mitoxantrone+ cytarabine; SC: supportive care; ATRA: all-trans-retinoic acid; ATO: arsenic trioxide; DA: daunorubicin+ cytarabine; DCAG: decitabine+ aclarubicin + cytarabine+ recombinant granulocyte colony-stimulating factor; IA: idarubicin+ cytarabine; IDA: idarubicin; DCA: Decitabine; Ara-C: cytarabine
